# A scoping review on barriers and facilitators to harm reduction care among youth in British Columbia, Canada

**DOI:** 10.1186/s12954-024-01063-1

**Published:** 2024-10-23

**Authors:** Kassey Beck, Katija Pallot, Michelle Amri

**Affiliations:** 1 Faculty of Health Sciences, Simon Fraser University, 8888 University Drive, Burnaby, BC V5A 1S6 Canada; 2 The W. Maurice Young Centre for Applied Ethics, School of Population and Public Health, University of British Columbia, 2206 East Mall, Vancouver, BC V6T 1Z3 Canada

**Keywords:** Harm reduction, Substance use, Substance abuse, Overdose, Young people who use drugs, Youth who use drugs, British Columbia, Canada

## Abstract

**Background:**

Progressive harm reduction policies have been implemented in British Columbia, Canada. However, youth who use drugs face barriers to receiving harm reduction care, resulting in increasing opioid-related hospitalizations and drug toxicity deaths. This scoping review collates peer-reviewed evidence to understand the barriers and facilitators faced by youth who use drugs when accessing harm reduction programming in British Columbia, Canada.

**Methods:**

This scoping review entailed conducting a systematic search of relevant databases to identify relevant articles. Articles were included if they: (i) contained youth falling between the ages of 12 and 26 years old; (ii) explored accessibility, barriers, and/or facilitators to harm reduction care or related topics; (iii) were empirical research articles using primary data (i.e., reviews, grey literature, theoretical or conceptual papers, books, etc. were excluded); and (iv) were available in the English language, given the geographic focus on British Columbia.

**Results:**

A total of 398 sources were identified and ultimately, data from 13 sources were charted and extracted. When investigating barriers to harm reduction care among youth, four themes emerged: self-stigma, service navigation, service delivery, and negative provider interactions. Furthermore, in exploring factors that facilitate harm reduction care for youth, four themes surfaced: ability to meet basic needs, positive provider interactions, social networks, and risk mitigation guidance.

**Conclusions:**

The expansion of harm reduction services in 2016 did not fully address accessibility challenges faced by youth who use drugs. Barriers continue to hinder harm reduction engagement, while supportive networks, positive provider interactions, and the ability to meet basic needs facilitated sustained access. Tailored policy interventions rooted in equity are crucial to improving access to harm reduction services for youth who use drugs.

**Supplementary Information:**

The online version contains supplementary material available at 10.1186/s12954-024-01063-1.

## Background

Harm reduction (HR) is an evidence-based strategy aimed at mitigating adverse health, social and legal impacts associated with substance use [1]. HR employs a spectrum of targeted interventions designed to help individuals meet their substance use goals without requiring strict abstinence. These interventions include strategies that promote safe drug use and minimize the risks associated with substance use, such as needle supply and exchange programs, supervised drug consumption sites, and take-home naloxone distribution [1, 2]. Additionally, HR initiatives can extend to reduced-use strategies and recovery support services, encompassing pharmacotherapies and low-barrier treatment programs to alleviate reliance on the toxic drug supply [1, 2].

Canada has supportive federal drug policies acknowledging the efficacy of HR with varying levels of adoption and implementation of the spectrum of HR initiatives between provinces and territories [3]. In particular, British Columbia (BC) is home to over half of the overdose prevention sites (OPS) operated nationwide and has historically been an early leader in progressive and novel HR approaches to addressing substance use related harm [4, 5]. Although youth across Canada may encounter similar barriers to accessing HR services, healthcare and social services delivery varies significantly between provinces. This study employs a single province approach to investigate the barriers youth face in accessing HR programs in the most progressive province in Canada and one of the most progressive jurisdictions in the world, BC.

In 2016, BC saw a mass expansion of HR initiatives, including OPS, following the provincial state of emergency declaration announced in response to an exponential increase in illicit drug-related deaths [4, 6]. Since then, other HR strategies have been implemented for people who use drugs, including increased offerings of opioid agonist therapy (OAT) modalities, low-barrier detox and treatment programs, needle and supply exchange programs, and more novel approaches like prescribed safer supply [1, 7]. Although findings from BC may offer insights for similar provinces, the substantial political and social variability across provinces may limit the generalizability of these results. For example, Alberta, a neighbouring province of BC, implements a recovery-based model and allocates funding towards more restrictive substance use care options [8, 9], highlighting the divergence in provincial approaches.

BC was also the first and only province in Canada to pilot an exemption to the Controlled Drugs and Substances Act [10]. In January 2023, this exemption decriminalized drug use and possession of small amounts of selected substances for personal consumption for people over 18 [10]. Decriminalization was initially implemented as a HR strategy in response to the toxic drug crisis, aiming to reduce stigma associated with substance use and facilitate access to essential social and health services for people who use drugs [10]. However, despite these intentions, numerous public health experts and people who use drugs voiced concerns and challenged the government's portrayal of decriminalization as a solution to a complex crisis [11–13]. They argued that this portrayal overlooked the continued reliance on an illicit and unregulated supply of drugs [11–13]. In April 2024, BC’s New Democratic Party Government which had applied for the historic exemption, rolled back on the policy, narrowing the applicability of decriminalization and recriminalizing the use of drugs in public spaces [14]. This reversal underscores the politicization and volatility of HR policies and programs in BC, emphasizing the heightened significance of scholarly research in this field and evidence-based solutions [15].

Despite an expansive scale-up of HR programming, many British Columbians, particularly youth, struggle to access such programs. Youth within BC substance use programming typically encompass individuals between the ages of 12 and 24, while some youth based programs will engage with youth up to 26 [16, 17]. Young people who use drugs (YPWUD) face several age-based barriers to accessing necessary HR measures, including the historical emphasis on abstinence-only care models within this demographic [18]. Age-restrictive policies have contributed to more adverse health outcomes for YWPUD, such as increased opioid-related hospitalizations [19]. In fact, the drug toxicity death rate for British Columbians under 19 has steadily climbed since 2019, increasing from 1.4 deaths per 100,000 to an all-time high of 3.8 deaths per 100,000 in 2022 [20]. Notably, illicit drug toxicity has become the leading cause of death for adolescents aged 10 to 18 and young adults aged 19 to 39 in BC [21]. The significant increase in overdose-related deaths in BC despite HR program expansion can be largely attributed to the unregulated toxic drug supply [22]. Additionally, there is a shortage of inhalation OPS options, which has become the most common route of substance use for people who use drugs in BC [23, 24].

Despite the available evidence surrounding barriers to HR programming offered to adults [25]—including other priority populations like pregnant or parenting people [26] and sex workers [27, 28]—a notable gap persists in our comprehensive understanding of the specific challenges faced by YPWUD [29]. Barriers experienced by adults who use drugs across BC include structural violence [26], criminalization and police-related factors [27, 28], stigma [25, 26, 30], long wait times [31], lack of information [32], distrust [30], and restrictive program policies [31]. These barriers contribute to the inaccessibility of HR services and a reluctance to seek care. Understanding the obstacles related to youth access to HR services is crucial, particularly when considering the disproportionate substance-related harms youth face [20]. Targeting youth with effective HR programming may lay the groundwork for reduced substance-related negative consequences later in life, particularly consequences pertaining to health and well-being [33].

This review answers the broad and guiding research question: What barriers and facilitators do YPWUD face when accessing harm reduction programming in BC, Canada? Given BC's comparatively wide availability of HR services, it is likely that youth in other regions may face barriers to accessing HR care on a grander scale. This review collates existing knowledge and novel insights to guide efforts to overcome barriers and continue actions for noted facilitators, particularly for community partners working on youth health and substance use interventions.

## Methods

A scoping review was used to systematically explore the breadth of peer-reviewed literature on the topic and identify existing gaps in knowledge. Given the emerging evidence concerning barriers to HR among youth [29], this form of knowledge synthesis is valuable for generating recommendations that inform future research, practice, and policies. HR programming for youth is a complex and evolving field, particularly with the expansion of HR initiatives since 2016. A scoping review is well-suited to capture the latest developments and emerging trends in this area, ensuring that policymakers can refer to a collection of studies that better represent the current state of the literature. A five-step approach based on established guidelines by Levac, Colquhoun, and O’Brien was applied [34]. The steps of: (i) identifying the research question; (ii) identifying relevant sources; (iii) selecting evidence; (iv) charting the data; and (v) collating, summarizing, and reporting will be discussed in more depth below. Reporting adheres to the standards outlined in the Preferred Reporting Items for Systematic Reviews and Meta-Analyses extension for Scoping Reviews (PRISMA-ScR) [35]. Given the nature of the scoping review drawing on published articles, ethical considerations related to participant consent did not apply. There is no protocol for this scoping review.

### Step 1: identifying the research question

The research question guiding this review asks: What barriers and facilitators do YPWUD face when accessing harm reduction programming in BC, Canada?

### Step 2: identifying relevant sources

With the guidance of a research librarian at Simon Fraser University, a search strategy was developed to identify relevant sources in the following databases: MEDLINE, PsycINFO, and Scopus. Table 1 contains the search string used for MEDLINE, but all search strings used in each database are available in the supplementary file. MEDLINE was selected for its strong focus on biomedical research [36], PsycINFO to capture the field of health psychology [37], and Scopus to encompass a broad spectrum of studies across life sciences, social sciences, physical sciences, and health sciences [38]. The initial search string was formulated in MEDLINE, employing text word and subject heading terms related to barriers, HR, and adolescents and/or youth to identify peer-reviewed journal articles. Studies published before 2016 were omitted, aligning with the contextual relevance of the province-wide state of emergency [6]. Additionally, reference lists of final sources charted were searched to identify any relevant sources not found through the database searches, but this did not result in the inclusion of additional final hits. Search results were uploaded to Covidence software [39], which automatically removed duplicates and facilitated the screening process. The search was completed on October 23, 2023, resulting in studies published after this date being excluded.

**Table 1:** MEDLINE search strategy

#	Search statement	Results
1	"youth" OR "adolescen*" OR "young adult"	2,889,106
2	"adolescent"[MESH]	2,226,545
3	("youth" OR "adolescen*" OR "young adult") OR ("adolescent"[MESH])	2,889,106
4	"substance-related disorders"[MESH]	312,992
5	"harm reduction"[MESH]	4191
6	"harm reduction" OR "overdose prevention" OR "supervised consumption" OR "injection site" or "opioid agonist"	29,618
7	(("harm reduction" OR "overdose prevention" OR "supervised consumption" OR "injection site" or "opioid agonist") OR ("harm reduction"[MESH])) OR ("substance-related disorders"[MESH])	334,652
8	"barrier*" OR "constraint*" OR "limitation*" OR "concern*" OR "access*" OR "experienc*"	3,622,444
9	"British Columbia*" OR "Vancouver"	138,492
10	(((("youth" OR "adolescen*" OR "young adult") OR ("adolescent"[MESH])) AND ((("harm reduction" OR "overdose prevention" OR "supervised consumption" OR "injection site" or "opioid agonist") OR ("harm reduction"[MESH])) OR ("substance-related disorders"[MESH]))) AND ("barrier*" OR "constraint*" OR "limitation*" OR "concern*" OR "access*" OR "experienc*")) AND ("British Columbia*" OR "Vancouver")	390
11	(((("youth" OR "adolescen*" OR "young adult") OR ("adolescent"[MESH])) AND ((("harm reduction" OR "overdose prevention" OR "supervised consumption" OR "injection site" or "opioid agonist") OR ("harm reduction"[MESH])) OR ("substance-related disorders"[MESH]))) AND ("barrier*" OR "constraint*" OR "limitation*" OR "concern*" OR "access*" OR "experienc*")) AND ("British Columbia*" OR "Vancouver") Filters: from 2016 – 2023	251

### Step 3: selecting evidence

**Fig. 1:**
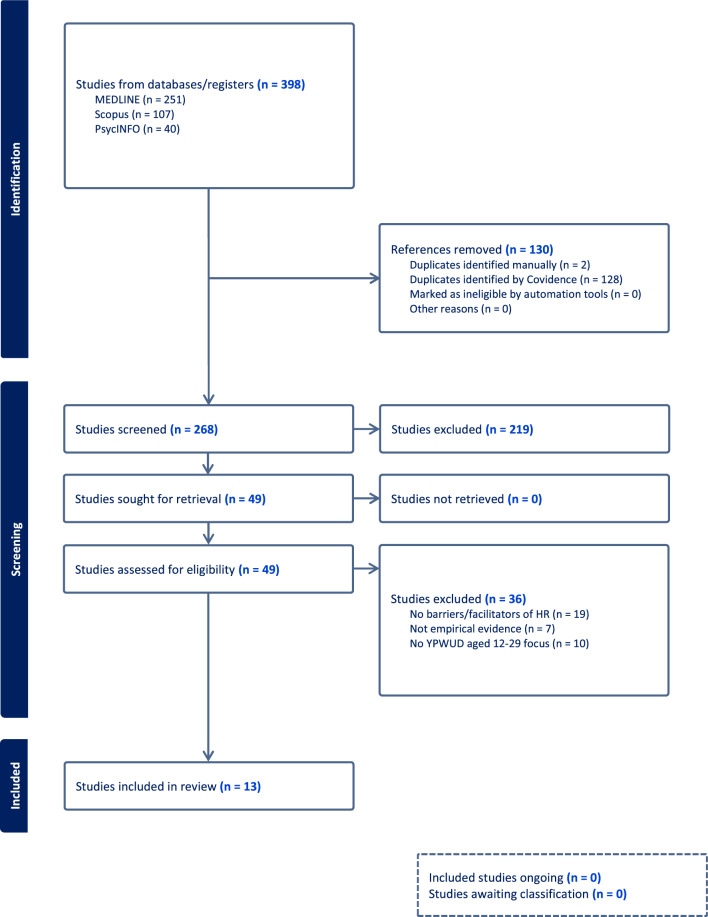
PRISMA flow diagram

Two independent researchers undertook a two-stage screening process to apply the inclusion criteria, first screening titles and abstracts and, subsequently, full texts that were screened during the first stage. This step-by-step process is illustrated through a PRISMA flow diagram (Fig. 1). Articles were included if they: (i) contained youth falling within the ages of 12 and 26 years old at the time of study enrolment to capture those currently engaged in or aging out of HR services; (ii) explored accessibility, barriers, and/or facilitators to harm reduction care or related topics; (iii) were empirical research articles using primary data (i.e., reviews, grey literature, theoretical or conceptual papers, books, etc. were excluded); and (iv) were available in the English language, given the geographic focus on BC. Any discrepancies were resolved through consensus meetings where the rationale for source inclusion was meaningfully discussed. This ensured a robust and well-justified selection process for the final inclusion of articles.

Although this scoping review focuses on populations between the ages of 12 and 26, we included one study that captured the perspectives of young people up to age 29. The research team justified this, as this study met inclusion criteria and explored barriers related to youth HR care and the transition to adult substance use services. Including this study was further rationalized due to the scarcity of evidence and its valuable insights into barriers to care. One study drawing from the experiences of youth service providers was included due to its substantial discussion of barriers and facilitators of youth accessing HR services [40]. Studies that explored barriers and/or facilitators to HR among a population of both youth and adults were excluded if they did not specifically explore youth-related aspects of care. For example, a study by Homayra et al. was excluded because it focused on patients up to 45 years old and did not specifically explore youth-related aspects of HR care [41]. Similarly, articles that detailed findings from BC in addition to other locations were included, but only information on BC was included in our scoping review. For instance, the study by Marchand et al. detailed findings from both BC and Alberta [42], but findings from Alberta were excluded, given the focus of this scoping review.

### Step 4: charting the data

A data extraction form was developed using Covidence software to facilitate the charting process for the final articles included in the study. Table 2 contains the details of these final articles by capturing the article title, author(s), year published, location(s) of study, study design, foci/us of study, age range, and key conclusion(s). To enhance reliability, the researchers independently extracted data from the first five studies, testing the data-charting form and assessing the consistency of their approach through meetings. The remaining articles were charted independently, and discrepancies were resolved through consensus meetings.

**Table 2:** Articles collated in the scoping review

Article title	Authors	Year published	Location(s) of study	Study design	Foci/us of study	Enrolment age range	Sample size (number of individuals)	Key conclusion(s)
Social-structural factors influencing periods of injection cessation among marginalized youth who inject drugs in Vancouver, Canada: An ethno-epidemiological study	Boyd J, Fast D, Hobbins M, McNeil R, Small W	2017	Vancouver	Qualitative	“How can we best reduce harms among youth who inject drugs as they move in and out of periods of injection cessation.”	14–26	22	“Periods of injection cessation were influenced by access to harm reduction-informed youth-focused services, transitions in route of administration, and the provision of housing and social supports.”
Still “at risk”: An examination of how street-involved young people understand, experience, and engage with “harm reduction” in Vancouver’s inner city	Bozinoff N, Small W, Long C, DeBeck K, Fast D	2017	Vancouver	Qualitative	“How do young people, who are involved in the ‘street drug scene’, understand, experience and engage with HR.”	14–26	13	“Young peoples’ multiple understandings, experiences, and engagements with HR in this setting illustrate the limitations of the existing infrastructure in improving their broader life chances.”
Inability to access addiction treatment predicts injection initiation among street-involved youth in a Canadian setting	DeBeck K, Kerr T, Nolan S, Dong H, Montaner J, Wood E	2016	Vancouver	Mixed methods	“Whether inability to access addiction treatment was associated with injection initiation among a cohort of street-involved youth.”	14–26	462	“Inability to access addiction treatment was common among the sample and associated with injection initiation.”
Risk mitigation guidance and safer supply prescribing among young people who use drugs in the context of COVID-19 and overdose emergencies	Giang K, Charlesworth R, Thulien M, Mulholland A, Barker B, Brar R, Pauly B, Fast D	2023	Vancouver	Qualitative	“How can lessons learned during the introduction of risk mitigation guidance and safer supply prescribing be applied to the development of more effective care programming for young people who use drugs, and particularly those who are experiencing street-involvement and have received a diagnosis of opioid use disorder.”	Youth aged 19–24 & addiction medicine providers offering care to youth	30 youth, 10 addiction medicine providers offering care to youth	“Findings underscore the importance of providing YPWUD with a safe supply of the substances they are actively using alongside a continuum of substance use treatment and care, and the need for both medical and community-based safe and safer supply models.”
“Getting out of downtown”: A longitudinal study of how street-entrenched youth attempt to exit an inner-city drug scene	Knight R, Fast D, DeBeck K, Shoveller J, Small W	2017	Vancouver	Qualitative	“Identify how young people envisioned exiting a local, inner-city drug scene in Vancouver, Canada, as well as the individual, social and structural factors that shaped their experiences.”	14–26	75	“Policies and interventions that can facilitate young people’s efforts to reduce engagement with Vancouver’s inner-city drug scene are critically needed, including educational and/or occupational training opportunities, ‘low threshold’ addiction services, and access to supportive housing.”
“We need to build a better bridge”: Findings from a multi-site qualitative analysis of opportunities for improving opioid treatment services for youth	Marchand K, Fogarty O, Pellatt KM, Vig K, Melnychuk J, Katan C, Khan F, Turuba R, Kongnetiman L, Tallon C, Fairbank J, Mathias S, Barbic S	2022	^a^BC urban, BC interior, AB northern, AB urban	Qualitative	“Identify youth-centered opportunities for improving opioid treatment services.”	16–29	11 (BC urban), 1 (BC interior)	“This study identifies fundamental directions for the operationalization and implementation of youth-centered opioid treatment services.”
“The system always undermined what I was trying to do as an individual”: Identifying opportunities to improve the delivery of opioid use services for youth from the perspective of service providers in four communities across British Columbia, Canada	Marchand K, Turuba R, Katan C, Fogarty O, Fairbank J, Tallon C, Mathias S, Barbic S	2023	Kelowna, Prince George, Vancouver, Victoria	Q ualitative	“Identify opportunities to improve the quality of opioid use services from the perspective of youth service providers.”	Service providers providing care to youth aged 16–24	41	“These findings underscore the need for a coordinated multi-level response: developing youth-specific standards, increasing inter-organizational activities and collaboration, and creating programs that are specific to youths’ needs.”
Navigating opioid agonist therapy among young people who use illicit opioids in Vancouver, Canada	Pilarinos A, Kwa Y, Joe R, Thulien M, Buxton JA, DeBeck K, Fast D	2022	Vancouver	Qualitative	“Explore how young people navigated opioid agonist therapy over time, including periods of engagement, disengagement, and avoidance.”	14–26	56	“Findings underscore the importance of working collaboratively with young people to develop treatment plans and timelines and suggest that OAT engagement and retention among young people could be improved by expanding access to OAT.”
Interest in using buprenorphine-naloxone among a prospective cohort of street-involved young people in Vancouver, Canada	Pilarinos A, Bingham B, Kwa Y, Joe R, Grant C, Fast D, Buxton JA, DeBeck K	2023	Vancouver	Quantitative	“Examine factors associated with initial buprenorphine-naloxone interest and the time to a positive change in buprenorphine-naloxone interest or enrollment, in addition to identifying reasons for buprenorphine-naloxone disinterest.”	14–26	281	“A need remains to improve the continuum of HR and treatment supports for adolescents and young adults.”
Methadone maintenance treatment discontinuation among young people who use opioids in Vancouver, Canada	Pilarinos A, Kwa Y, Joe R, Dong H, Grant C, Fast D, Buxton JA, DeBeck K	2023	Vancouver	Quantitative	“Identify factors associated with methadone maintenance treatment discontinuation among adolescents and young adults.”	16–24	160	“Efforts to revise methadone maintenance treatment programming may improve treatment retention and reduce toxic drug fatalities.”
Navigating treatment in the shadow of the overdose crisis: Perspectives of youth experiencing street-involvement across British Columbia	Thulien M, Charlesworth R, Anderson H, Dykeman R, Kincaid K, Sedgemore K, Knight R, Fast D	2022	Kelowna, Prince George, and Vancouver	Qualitative	“Explore youth access to integrated mental health and substance use treatment across the province.”	14–24	63	“Findings point to the inability of existing services and systems to address entrenched marginalization and chronic instability, and underscore the importance of relationship-, trust-, and future-building to providing treatment and care to youth.”
A qualitative study exploring how young people perceive and experience substance use services in British Columbia, Canada	Turuba R, Amarasekera A, Howard AM, Brockmann V, Tallon C, Irving S, Mathias S, Henderson J, Marchand K, Barbic S	2022	Province-wide	Qualitative	“Explore how youth perceive and experience substance use services in British Columbia, Canada.”	16–24	30	“Results suggest a clear need to prioritize substance use prevention and early interventions specifically targeting youth and young adults.”
“A peer support worker can really be there supporting the youth throughout the whole process”: A qualitative study exploring the role of peer support in providing substance use services to youth	Turuba R, Toddington C, Tymoschuk M, Amarasekera A, Howard AM, Brockmann V, Tallon C, Irving S, Mathias S, Henderson JL, Barbic S	2023	Province-wide	Qualitative	“Explored the role of peer support in providing substance use services to youth in British Columbia and how best to support them in their role.”	Peers working with youth aged 12–24	18	“Findings from this study call for improved integration of peer support into service environments, as well as standardized training that is in-depth and continuous.”

^a^Our analysis excluded results from northern and urban Alberta communities due to the focus of our study

### Step 5: collating, summarizing, and reporting the results

Thematic analysis—a qualitative technique utilized for recognizing, examining, and structuring emerging patterns within the gathered data [43]—was employed to derive themes pertaining to barriers and facilitators. A deductive, data-driven analysis with no predefined codes was used to identify emergent themes and sub-themes in alignment with the research question. The researchers initiated the process by independently familiarizing themselves with the data and documenting preliminary reflections on potential codes and themes. Following this process, researchers independently coded the data and grouped similar codes into several sub-themes. Through discussion, these sub-themes were combined to identify eight broader themes to capture the interplay and complexity of identified barriers and facilitators.

## Results

The database searches produced 398 articles, from which 130 duplicates were removed. A total of 268 titles and abstracts were screened, and 49 documents met the inclusion criteria and were retrieved for full-text review. After reviewing the full texts, 13 articles were extracted for this scoping review (Table 2).

### Description of articles

Of the articles included in this review, ten employed qualitative analysis, two employed quantitative analysis, and one used mixed methods. Qualitative studies highlighted the perspectives of YPWUD, while quantitative studies illustrated reasons for HR discontinuation or avoidance and the prevalence of each within a sample. Sample sizes ranged from 11 to 75 participants for the qualitative studies, 160 to 281 for the quantitative studies, and 462 participants in the mixed methods study.

Participant age ranges varied between studies and age reporting was inconsistent, as some studies reported a median and interquartile range (n = 5) [44–48], whereas others listed explicit age ranges (n = 9) [42, 44, 45, 49–54]. For the studies that reported a median age, all were greater than 21. For those with explicit age ranges, two studies did not include perspectives of youth younger than 19 [52, 54]. However, we feel it is notable that only two studies contained perspectives of youth aged 12 to 14 [44, 45].

Geographically, most included studies focused on Vancouver, BC (n = 11). Among these, three studies were conducted at multiple sites spanning Kelowna, Prince George, Victoria, and an undisclosed city in the BC interior, in addition to Vancouver (for more details on studies, please see Table 2). Additionally, two studies included participants from across the entire province [44, 45].

Recruitment strategies varied across studies included in this review. Many drew participants from the at risk youth study [46–52]. Initial recruitment strategies for this study included techniques for reaching hidden populations, including extensive street-based outreach throughout different neighbourhoods, nighttime outreach, and snowball sampling [52]. To draw specific participants from this study, some researchers employed purposive recruitment via staff phone calls, emails, and social media messages [51, 52, 54]. Additional recruitment of street-involved youth targeted local drug treatment settings, drop-in centers, shelters, and other support services (e.g., recreational activities, food, harm reduction, medical care, and housing and social assistance) [49, 53]. Studies also drew participants through staff, social media pages, and targeted advertisements from Foundry [45, 54], a youth-dedicated primary care center operating across BC [16]. Community-based partners and other organizations serving youth, such as mental health services, schools, and community centers were also contacted to provide recruitment adverts [42, 45]. Service providers were recruited with the support of provincial and community-based partners, who distributed information about the study within their agencies and throughout their wider networks [40]. Peer support workers were recruited through social media, targeted advertisements, and organizations that provide peer support services across the province [44].

### Identified themes

Eight themes emerged from the thematic analysis of the barriers and facilitators of HR programming among youth in BC. Barriers were self-stigma (n = 5), service navigation (n = 11), service delivery (n = 11), and negative provider interactions (n = 7). Facilitators were ability to meet basic needs (n = 4), positive provider interactions (n = 4), social networks (n = 4), and risk mitigation guidance (n = 1). An overview of these themes and sub-themes, where applicable, are presented in Table 3, and a depiction of themes that appear in each article can be found in Table 4.

**Table 3:** Barrier and facilitator themes and sub-themes

Barrier themes				
Themes	Self-stigma	Service navigation	Service delivery	Negative provider interactions
Sub-themes	N/A	Lack of information or transparency	Waitlists	Discrimination
		Lack of service options	Cost	Distrust
		Transportation /location	Hours of operation	Lack of autonomy
		Lack of tailored services	Limited capacity	
			Age restrictions	
			Program restrictions	

Facilitator themes				
Themes	Ability to meet basic needs	Positive provider interactions	Social networks	Risk mitigation guidance

**Table 4:** Depiction of themes that appear across articles

Study title [reference]	Themes							
	Barriers				Facilitators			
	Self-stigma	Service navigation	Service delivery	Negative provider interactions	Ability to meet basic needs	Positive provider interactions	Social networks	Risk mitigation guidance
Social-structural factors influencing periods of injection cessation among marginalized youth who inject drugs in Vancouver, Canada: An ethno-epidemiological study [52]		X	X		X	X	X	
Still “at risk”: An examination of how street-involved young people understand, experience, and engage with “harm reduction” in Vancouver’s inner city [50]	X	X	X					
Inability to access addiction treatment predicts injection initiation among street-involved youth in a Canadian setting [48]		X	X		X			
Risk mitigation guidance and safer supply prescribing among young people who use drugs in the context of COVID-19 and overdose emergencies [54]			X	X				X
“Getting out of downtown”: a longitudinal study of how street-entrenched youth attempt to exit an inner-city drug scene [51]		X	X		X		X	
Navigating treatment in the shadow of the overdose crisis: Perspectives of youth experiencing street-involvement across British Columbia [53]		X		X				
“We need to build a better bridge”: Findings from a multi-site qualitative analysis of opportunities for improving opioid treatment services for youth [42]	X	X	X	X		X		
“The system always undermined what I was trying to do as an individual”: Identifying opportunities to improve the delivery of opioid use services for youth from the perspective of service providers in four communities across British Columbia, Canada [40]	X	X	X					
Navigating opioid agonist therapy among young people who use illicit opioids in Vancouver, Canada [49]			X	X				
Interest in using buprenorphine-naloxone among a prospective cohort of street-involved young people in Vancouver, Canada [47]		X		X				
Methadone maintenance treatment discontinuation among young people who use opioids in Vancouver, Canada [46]		X	X					
A qualitative study exploring how young people perceive and experience substance use services in British Columbia, Canada [45]	X	X	X	X		X	X	
“A peer support worker can really be there supporting the youth throughout the whole process”: A qualitative study exploring the role of peer support in providing substance use services to youth [44]	X	X	X	X	X	X	X	

### Barriers

#### (1) Self-stigma

The use of HR services among YPWUD was notably affected by the pervasive influence of stigma, particularly self-stigma, which involves individuals internalizing society's negative perception of substance use and integrating it into their personal beliefs and self-perception [40, 42, 44, 45, 50, 55]. Instances included feelings of shame, embarrassment, or disappointment about their substance use [42, 44, 45, 50], fear of invalidation [40], and fear of rejection or judgment from the community, family members, and service providers [40, 44]. YPWUD expressed concerns about how their substance use might be perceived by others, including providers, and did not want to be stereotyped as an “addict,” a “bad person,” or a “criminal” [45 p.4]. These youth advocated for having information more widely available to help reduce societal stigma by increasing awareness of substance use [45]. These findings underscore the impacts of internalized stigma on the willingness of youth to access essential HR services.

#### (2) Service navigation

Service navigation, specifically the ability to access available and appropriate services equitably, was identified as a barrier to youth accessing HR programs. The exploration of this barrier encompassed the following sub-themes: a lack of information or transparency, lack of service options, location of services, and a lack of tailored services.

##### (2.i) Lack of information or transparency

Although only identified across three studies, youth navigating HR services expressed a lack of accessible information regarding available resources and confusion about the appropriate places to access support [44, 45, 51]. Turuba et al. highlighted that many youth were unaware of where to access services, often assuming that all available options were abstinence-based, which many found to be too intimidating [45]. Confusion surrounding conflicting options and approaches to OAT for the treatment of opioid use disorder across various programs and providers was a significant concern [51]. Youth emphasized that readily available access to information could help to address other barriers to care, such as stigma [44, 45]. They highlighted the need for widespread availability of information, such as comprehensive educational materials available in schools [44, 45]. These resources should encompass factors influencing youth substance use behaviours and the long-term impacts of substance use [45].

##### (2.ii) Lack of service options

Across the spectrum of HR service provision, youth experienced limited service options. There was a lack of youth-specific care options [40], adequate low-barrier programming [42], and sufficient preventive care [44]. Youth stressed the importance of enhancing accessibility to HR services, including drug-checking initiatives, risk mitigation through safe/safer supply prescribing, lower-barrier evidence-based treatment, and detox centers that incorporate HR practices [40]. Additionally, service providers expressed the necessity for dedicated support for youth engaged in stimulant use [40]. Youth also explicitly described how OAT options are presented as overly restrictive [46, 47]. Sometimes, providers only offered buprenorphine-naloxone despite youth explicitly requesting other OAT modalities like methadone [49]. These service option limitations highlight the pressing need for expanded, accessible, and youth-tailored interventions to address diverse needs and preferences within the continuum of care.

##### (2.iii) Location of services

The location of available services for youth was identified as a logistical barrier to accessing adequate HR for YPWUD [40, 44, 45, 48, 50]. For some youth, accessing these services necessitated travel outside of their home communities. However, many emphasized that they had no adequate form of transportation [40, 42, 45, 50]. Even when youth had relatively better access to services, this barrier persisted as they hesitated to visit areas they found uncomfortable or actively sought to avoid, like Vancouver's Downtown Eastside [40, 50]. Despite the concentration of HR services in this area, the associated stigma acted as a significant deterrent for Vancouver's youth. This heightened stigma made it even more challenging for youth to access HR services, as there are minimal services elsewhere, and these alternatives are often resource constrained. The barrier of distant service locations underscores the importance of more widely accessible HR services for YPWUD provincewide.

##### (2.iv) Lack of tailored services

The final service navigation barrier explored was limited specialized service options for intersections of youth. Youth highlighted the inability to find culturally appropriate services within the health and social systems along the HR continuum [40, 44, 45, 47, 52, 53]. Youth advocated for more culturally safe spaces for Indigenous YPWUD, in addition to staff who have adequate cultural sensitivity and humility training to address this gap [44, 47]. Care and spaces specific to addressing the needs of gender and sexually diverse youth were another highlighted gap experienced within HR service provision [40, 45, 52]. Youth illuminated a lack of trauma-informed practices, specifically within acute care and emergency services, which led to resistance to accessing any care and support [53]. This resistance also saw youth avoid social support services like shelters and case management teams for fear that their substance use would be reported to child protective services [53]. The identified service navigation barrier strengthens the urgent need for more tailored services to enhance accessibility within the HR continuum of care.

#### (3) Service delivery

Service delivery emerged as a substantial barrier for YPWUD in BC. Service delivery encompasses system- and program-specific barriers or restrictions to youth access to HR services. Within the broader theme of service delivery, several sub-themes contribute to the complexity of this barrier, which are explored below. Subthemes include wait times, cost, hours of operation, limited capacity, age restrictions, and program restrictions.

##### (3.i) Wait times

Youth described experiencing wait times across the full spectrum of HR service provision. This included waiting for treatment, referrals, and other specialized care options [40, 42, 44, 45, 48, 51, 52]. Additionally, youth highlighted having to wait for their OAT provider to write a prescription in community clinics and additional time waiting for OAT prescriptions at pharmacies [42]. Wait times undermined readiness to engage in care for participants and ultimately posed a barrier for youth accessing HR services.

##### (3.ii) Cost

Another barrier to accessing HR explored by YPWUD was the cost of programming [42, 45–47, 51, 52], including daily dispensing fees [51, 52], treatment [42], and travel costs [42]. An additional barrier was the inability to keep subsidized housing, resulting from attending ministry-funded residential treatment facilities [50]. These financial burdens serve to compound the challenges faced by YPWUD, thereby presenting a multifaceted barrier to their access to HR services.

##### (3.iii) Hours of operation

The operational hours of HR programs were presented as an additional obstacle to youth access. This included a lack of extended evening and nighttime hours and weekend availability at pharmacies [46, 49], as well as clinical programs [40, 42, 45, 48]. Restricted hours of operation emerged as a barrier to youth accessing HR, highlighting the urgent need for expanded and more flexible service hours.

##### (3.iv) Limited capacity

Youth and providers alike discussed a lack of capacity within the HR care provision system. In one instance, a patient was removed from their physician’s patient list due to an overwhelming caseload [52]. Healthcare workers acknowledged an inefficient use of time and resources, inconsistencies between providers, limited trust and confidence in service partnerships, and poor care continuity within the system [40]. Some service providers and peers explained that they received minimal support from their organizations and did not always have access to training that highlighted best practices [40, 44]. The identified lack of capacity within HR care provision underscores the pressing need for more and better resources, training, and support mechanisms within the youth-focused HR framework.

##### (3.v) Age restrictions

YPWUD experienced age-related restrictions as a barrier to HR services. Specific experiences of these restrictions were seen in residential treatment, detox centers, and other community-based programming [48, 50, 54]. Providers expressed hesitancy to prescribe risk mitigation guidance safer supply to youth, especially those under the age of majority [54]. Risk mitigation guidance, introduced in 2020, represents a COVID-19-specific HR strategy intended to decrease the risk of overdose and withdrawal during a period when people were being asked to self-isolate [56, 57]. For those who are actively using substances, risk mitigation guidance allows for the prescription of a limited range of controlled, prescription medications such as 12-h sustained-release oral morphine (brand name M-Eslon®), hydromorphone tablets (brand name Dilaudid®), methylphenidate (brand name Ritalin®), dextroamphetamine sulphate tablets (brand name Dexedrine®), and benzodiazepine tablets (e.g., clonazepam). Expansion of this prescribing practice was not actualized for many youth across the province despite meeting criteria and explicit requests [54]. Selected studies also highlighted a gap in care provision when aging out of youth services, which is typically experienced by youth between the ages of 18 and 24 [42, 51, 52]. During this period, youth are shifting from youth-specific programming to adult-focused programming [42, 44, 49, 51, 52]. This shift was seen by youth as a barrier to accessing HR services due to an incomplete integration of systems. Youth aging out who did not successfully transition to adult services were more likely to disengage in OAT [51]. Age-related restrictions in various care settings collectively hinder fulsome access to HR services for youth.

##### (3.vi) Program restrictions

Program expectations or criteria dictated eligibility to access and ability to maintain engagement in HR services. Youth highlighted that certain HR programs presented "unrealistic or overwhelming expectations" [p. 35] — such as requiring them to search for employment while undergoing treatment — which hindered both their initial access to and sustained involvement in these services [50]. Overwhelmingly, studies emphasized that abstinence-based and zero-tolerance programming posed a barrier to youth accessing support for substance use [40, 44, 45, 49, 52]. Although sobriety can be a goal, abstinence-based and zero-tolerance programs are often intimidating, overly restrictive, and fail to meet the diverse needs of youth [45, 52]. These negative experiences were common, leading many youth to avoid the healthcare system and substance use programming altogether and to believe that cessation must be achieved independently [52]. Emphasizing strengths and achievements and setting realistic goals within HR programs empowered youth to navigate their expectations and external pressures, acknowledging that abstinence might not align with their objectives [45, 52]. One youth participant explained that they were encouraged to titrate off a stable buprenorphine-naloxone dose while receiving residential treatment despite that not being their recovery goal [49]. This highlights that, “not everybody wants to stop using substances” [p.5], and that recovery-oriented programming should allow for more flexibility when it comes to HR approaches to substance use [44]. Other logistical restrictions were experienced by youth, including being unable to complete the necessary paperwork to engage in care and services [48], and being turned away or discharged due to behavioural concerns [42]. Another barrier identified was the difficulty for youth to maintain their OAT due to restrictive missed dose policies and minimal options for take-home dosing of OAT [46, 49]. Program expectations and unrealistic criteria, especially abstinence-based approaches, pose barriers to both access and sustained youth engagement in HR services.

#### (4) Negative provider interactions

Negative provider interactions were a common barrier identified by youth. This included discrimination, distrust, and a lack of autonomy.

##### (4.i) Discrimination

Negative provider interactions were a notable barrier for youth seeking HR services [42, 44, 45, 47, 49, 53]. Interactions encompassed instances of explicit racism or discrimination [44, 45, 53], judgement [42, 44], negative or disrespectful treatment [45, 47], and frustration [49], which led youth to avoid or discontinue accessing HR supports. Negative experiences were described as common and dominant [42]. Youth also felt that their issues were not taken seriously and were often dismissed because they did not fit the stereotypical image of substance dependence, such as dropping out of school or having a poor home life [45]. Thulien et al. found that a vast majority of Indigenous youth described experiencing racism while seeking care [53].

##### (4.ii) Distrust

Studies also highlighted the effects of distrust of healthcare providers, which can result from previous negative experiences within the health and social system, preventing many participants from utilizing HR services [53, 54]. The fear of information sharing between different health and social services without consent was presented by youth, resulting in apprehension to seek care [53]. Similarly, youth were extremely wary of surveillance and information sharing inherent in case management teams, fearing potential judgment based on the personal details contained in their case files [53]. Indigenous youth, in particular, feared that accessing any kind of help could precipitate child protective services involvement and possible removal from their families of origin [53]. Some participants expressed reluctance to engage in peer support services because of concerns that the providers might lack formal training in substance use [45]. Distrust in healthcare providers holds significant importance as it erodes the foundation of patient-provider relationships, impedes effective communication, and diminishes adherence to care.

##### (4.iii) Lack of autonomy

Although only highlighted in two studies, lack of control over care was another factor identified by youth [44, 45]. Participants described being treated as if their age rendered them incapable of making wise decisions, with providers often undermining their role in decision-making [45]. These paternalistic interactions prevented youth from seeking further support [45]. Peer support workers emphasized the importance of letting youth “take the lead” [44 p.5], such as directing them to a range of support and deciding if or when it was appropriate to involve their families. This was a crucial way to sustain relationships with patients [44]. Lack of control while navigating substance use support can cause youth to disengage from the healthcare system.

### Facilitators

In addition to barriers, several facilitators to the accessibility of HR services were identified: ability to meet basic needs, positive provider interactions, social networks, and risk mitigation guidance.

#### (1) Ability to meet basic needs

Studies revealed the importance of addressing basic needs, such as housing, to facilitate access and utilization of HR services [44, 51, 52]. Access to housing was crucial for youth in initiating and maintaining drug injection cessation, highlighting the importance of meeting basic needs before exploring HR options [51]. Knight et al. illustrated the bureaucratic constraints of the medical system for YPWUD experiencing homelessness [51]. To access government assistance for the dispensing fees of methadone maintenance treatment, people need to qualify for social assistance. However, to qualify for social assistance, individuals need to provide a legal address. Like many young people experiencing homelessness, participants highlighted that they would rather sleep outside than in neglected and potentially dangerous single occupancy hotels [51]. The provision of necessities, encompassing shelter, stable housing, employment opportunities, clothing, hygiene facilities, and food, significantly benefited YPWUD in their engagement with HR services [48]. From the perspective of peer support workers, a holistic approach that addresses these foundational needs emerged as an integral component of HR initiatives and connected YPWUD to other supports [44].

#### (2) Positive provider interactions

A factor facilitating the utilization of HR among YPWUD was the presence of positive engagements with healthcare providers [42, 44, 52]. Studies illuminated that positive interactions with healthcare providers bolstered participants’ resilience and fortified their willingness to engage with HR services [42, 44, 52]. Positive interactions enabled youth to trust providers, persevere through challenges, and build self-confidence [42]. Having a service provider who took additional steps to support YPWUD, such as providing rides, meeting them in preferred settings, and checking in with them regularly, made youth feel genuinely cared for and increased their likelihood of returning [45]. The scarcity of positive provider interactions emphasizes the necessity for improved quality in interactions between youth and providers throughout the HR care continuum [44].

#### (3) Social networks

A common thread throughout studies included in this review was the influence of social support from friends and family on participants’ ability to access HR services and maintain reduced substance use [44, 51, 52]. For instance, a participant explained that financial assistance from their father was a determining factor in their ability to gain access to methadone maintenance treatment [52]. Another participant highlighted that family support was more effective than the services available to them in maintaining injection cessation [52]. Peer support workers identified support systems as integral aspects of holistic care [44]. However, although youth described these relationships as “game changers” [44 p.5], the widespread stigma of substance use often affected their ability to leverage these relationships for support [44, 51].

#### (4) Risk mitigation guidance

One study specifically explored the relationship between risk mitigation guidance prescriptions and concurrent OAT engagement. This study found that risk mitigation guidance prescriptions of hydromorphone could be particularly helpful for YPWUD who were interested in engaging with OAT [54]. Numerous prescribers emphasized the potential benefits of including hydromorphone tablets in conjunction with methadone or buprenorphine-naloxone treatments. This combined approach can alleviate initial cravings and withdrawal symptoms while allowing for a gradual adjustment to a stable therapeutic dose of OAT. Combined therapy fosters ongoing connections to care as YPWUD engage and re-engage with OAT over time. YPWUD were more likely to consider starting, restarting, and continuing with OAT if they could simultaneously access hydromorphone prescriptions [54].

## Discussion

To our knowledge, this is the first scoping review identifying the barriers and facilitators associated with accessing HR services among youth aged 12–26 in BC. This study captured perspectives from YPWUD, providers, and peer support workers. Synthesis across all sources identified four key barriers that reflect important features of youth HR care (self-stigma, service navigation, service delivery, and negative provider interactions) and four key facilitators (ability to meet basic needs, positive provider interactions, social networks, and risk mitigation guidance). Due to the dearth of evidence surrounding this topic, the barriers and facilitators unearthed in this study hold valuable insights that can guide the development of interventions and improvements to existing efforts. Findings from this study that coincided with the barriers experienced by adults were expected, such as stigma [25, 30], wait times [31], lack of information [32], distrust [30], and program restrictions [31]. We also expected that a lack of tailored services for youth and intersections of their identities impacted the ability of youth to meaningfully engage across the HR continuum [58]. This finding is supported by similar research on intersections of adults and their barriers to accessing HR programming [31].

There were also a few findings that were specific to youth. Although we expected age-based restrictions to be a prevalent theme, the aging out process that impacted the ability of youth to engage in HR services was unexpected. Additionally, given that HR services are primarily designed by and for adults [59], we anticipated reading about concerted efforts to improve access for younger adults transitioning into adult programming, yet this did not materialize. And lastly, an inability of youth to make autonomous decisions about their care was pervasive within the findings. However, this finding was expected given that it is featured in research on youth mental health care [60].

The stigma associated with the opioid crisis is far-reaching and significantly impacts the use of HR services among YPWUD. This stigma, particularly self-stigma, influences youth and their willingness to seek help [40, 42, 44, 45, 50]. Studies demonstrated widespread stigma is linked to provider discrimination [42, 44, 45, 47, 49, 53], limited access to information [44, 45], the location of services (e.g., Vancouver’s Downtown Eastside) [40, 50], and the inability of YPWUD to leverage social networks for support [44, 51], further exacerbating challenges in youth accessing HR services [44, 45, 51]. The findings highlight the profound impact of internalized stigma on youths' willingness to access essential HR services, consistent with prior research demonstrating that stigma acts as a barrier to healthcare utilization, especially mental health and substance use services, among various populations [30, 61–65].

It is imperative to dissolve the deeply rooted societal stigma of substance use, particularly among youth, to ensure access to necessary support and services without fear of judgment or discrimination. Youth themselves identified several actions to address stigma, including making information more widely available, incorporating appropriate substance use education in schools and for medical professionals in training, and investing in de-stigmatization campaigns [44, 45]. We echo their voices and stress the importance of prioritizing and implementing the following policy recommendations to further reduce stigma, as these measures promote a more informed, compassionate, and supportive approach to addressing substance use issues.

### Policy recommendations

The following section focuses on actionable policy recommendations to enhance HR service access for youth provincewide: service location accessibility, seamless youth-to-adult program transitions, advocating against zero-tolerance approaches in HR, integrating tailored practices into HR care, and fostering positive interactions between providers and youth.

### Location accessibility of services

Our scoping review revealed existing location and service navigation barriers impeding youth access to HR services in BC [40, 44, 45, 48, 50]. The broader literature supports that the location of services contributes to adverse health outcomes for all people who use drugs, not exclusively youth [66, 67]. In response to insights provided by the Office of the Representative for Children and Youth in BC report titled “Time to Listen: Youth Voices on Substance Use” from November 2018 [68], efforts were undertaken at the provincial level. BC expanded youth-based integrated child and youth care through Foundry, a partnership between BC Children’s Hospital, the Government of BC, the Graham Boeckh Foundation, the Michael Smith Foundation for Health Research, Providence Health Care and St. Paul’s Foundation [16, 68]. Youth in 16 communities across BC and within all five major regional health authorities have access to substance use and mental health services through this program [68]. Foundry’s expansion has improved access for youth, particularly in the lower mainland, where half of their current service sites exist. To address accessibility concerns outside this area, further expansion includes embedding integrated child and youth services into nine more communities. Seven of these are located in more rural and remote regions across BC [16]. This represents a promising start to improved accessibility to HR for youth in BC. However, fundamental gaps persist within the full spectrum of care within the province. These include embedding youth programming within regional health authorities (external to integrated child and youth offerings) and improving rural access to HR services [69]. Youth living in remote areas without age-specific HR services often must personally cover the cost of travelling outside their communities [70]. This financial barrier is particularly relevant for Indigenous youth, especially girls and women, as this population is more likely to resort to unsafe transportation practices, which significantly elevates their risk of experiencing violence [70].

To address these gaps, we propose mandating regional health authorities to broaden the provision of HR services, such as fulsome OAT options or overdose prevention access for youth through existing pathways of care when dedicated youth-based programs are unavailable. We recommend that regional health authorities assume responsibility for covering transportation costs for rural and remote youth or supporting youth to navigate existing resources. This would manifest as either direct health authority funding or leveraging the existing provincial Travel Assistance Program (TAP) to support youth in communities that lack readily accessible HR services [71]. Additionally, supporting BC status First Nations youth to apply for and access the medical transportation benefit offered by the First Nations Health Authority [72]. Finally, this might also involve integrating episodic overdose prevention services into existing health sites across the province, ensuring accessibility for youth in accordance with the BC Centre for Disease Control episodic overdose prevention service guidelines [73].

### Restrictive programming

Findings from our review revealed that youth viewed zero-tolerance, abstinence-based programming as an overarching barrier to HR service access [40, 45, 49, 52]. The conventional model of care for addressing youth substance use has focused primarily on abstinence [74]. The focus of treatment to eradicate substance use among youth has resulted in youth being distrustful and fearful of the current system of care [29, 75, 76]. An additional consideration is the lack of regulation of residential treatment facilities in BC [22, 77, 78]. There are no best practice guidelines nor formal evaluation processes for recovery-oriented programming in BC [22, 77, 78]. Understanding the efficacy of residential treatment is hindered by the limited availability of accessible evidence [78]. Additionally, there are regulatory and best practice concerns, particularly when compared to treatment pathways for well-established long-term health concerns like asthma and diabetes. These concerns underscore the importance of mandating and embedding evidence-based or -informed strategies along the continuum of care to minimize immediate and ongoing harm to YPWUD in BC. Broader evidence supports treatment programs that implore HR strategies like OAT prescribing and continuation of OAT to help prevent relapse and mitigate unintended consequences of abstinence like overdose [79, 80]. According to Spithoff et al., those who were engaged in OAT had lower relapse rates and better treatment outcomes than those enrolled in more restrictive programs [79].

Additionally, youth have advocated for more robust low-barrier HR options and progressive youth programming [40, 42, 47]. Safe and safer supply options aimed at mitigating the exponential death rate due to drug toxicity have recently gained traction in research and organizational documents, like the BC Coroner Service Death Review Panel [22, 58, 74, 77, 78]. Despite the essential discourse on regulated safe supply, youth have often been left out of these larger conversations [58]. It is crucial that YPWUD are not only included in such conversations, but also afforded fair consideration and opportunity to shape policies and strategies around drug use.

We recommend provincially funded and organized treatment programs that implore evidence-based care options and are robustly regulated, evaluated, age-appropriate, and timely as part of the spectrum of HR. Similarly, we support the utilization of OAT and other risk mitigation guidance prescribing within recovery-oriented programming as part of the HR spectrum of care to prevent relapse and consequential overdose for YPWUD. Finally, we add to the ever-growing body of work advocating for a safer supply of drugs that is accessible to all people at risk of death, including YPWUD [22, 29, 58, 77].

### Youth- to adult-based service transition

Our review highlights gaps in care experienced by youth when transitioning out of youth-based programs [42, 44, 46, 47, 49, 51, 52]. The primary BC provider of youth-specific substance use care is Foundry, serving individuals aged 12–24 [16]. Individuals who are 24 will age out of Foundry's care and transition to adult-based care [16]. For some youth, an unsuccessful transition in care led to disengagement from HR services altogether [51]. Youth-based advocacy surrounding this topic urges programs to engage with participants about readiness to transition to adult services, giving ample warning and time to build a meaningful connection with their new team or service provider [58]. Youth also highlighted that former youth-based program participants should not be denied access when they want to return for supplies or food [58]. The BC Centre on Substance Use guidelines for opioid use disorder include language around the importance of fostering connections to services for youth aging out of care, explicitly recommending that participants well-engaged in care with youth-based teams should continue to receive this care beyond the age cut-off [81]. The BC Centre on Substance Use also stated that before transitioning to adult programming, youth care teams should support youth to foster relationships with new teams, programs, and service providers [81]. This entails allocating an appropriate amount of time and ensuring the adequacy of services to enhance continuity of care. These findings align with existing research on transitions in care among youth aging out of mental health services [82]. Cleverley et al. found that youth should be actively involved and engaged in developing their transition plan to ensure engagement post-transition [82]. It was also suggested that, depending on readiness, transition care planning should begin a minimum of six months prior to transition and should include an established connection to adult services [82].

We recommend policy changes to age restrictions for youth-specific care to match emerging research and youth commentary about this barrier. We suggest flexibility in the aging out timeline for youth engaged in care. This flexibility would entail a transition period window and encourage teams to refrain from endorsing a strict age for transition. We also suggest an improved integrated transition system for youth who show readiness to transition to adult-based HR services. This transition system should incorporate input from individuals with lived and living experiences to ensure appropriateness [29].

### Culturally appropriate, gender-responsive, trauma-informed care

The scoping review identified a deficiency in culturally safe and Indigenous-led HR options for youth [40, 44, 45, 47, 52, 53]. A recent report found that out of 408 youth substance use services inventoried across the province, only 11 reported that they are Indigenous-only services [70], forcing patients to access services that are not culturally appropriate. In addition, one study found that despite a devoted facility located at a Vancouver hospital for cultural purposes (smudging, drumming, etc.), not one participant mentioned the use or awareness of the space [83]. Upon further investigation, the space was consistently locked and only accessible by security and a few designated staff [83]. Similarly, youth highlighted the dearth of gender-sensitive supports for both men and women, as well as inclusive services for gender and sexually diverse patients [40, 42, 45, 52]. There is currently a lack of tailored gender and sexually diverse substance use services, and mainstream services in BC are not designed to meet the needs of this population [29, 70]. This is notable as gender and sexually diverse youth exhibit significantly higher rates of substance use and poor health outcomes compared to their cisgender and heterosexual counterparts [84].

We recommend that regional health authorities provide adequate funding and support to expand tailored services for Indigenous and gender and sexually diverse YPWUD. This includes specific spaces that are designed by and for the communities that they exist to serve.

Youth expressed a desire for their service providers to consider the factors contributing to their substance use patterns, particularly trauma [44, 53]. Distrust and avoidance of health and social services tend to develop among youth who have experienced continuous institutionalization [85]. Trauma-informed practices strive to improve engagement in care and services by creating safe and respectful environments with a larger goal of avoiding further trauma [86]. In BC, Indigenous children constitute less than 10% of the child population but make up 68% of those in provincial care [87]. Thulien et al. noted that the reluctance of Indigenous youth to seek HR care is driven by fears of getting involved with child protective services and potential apprehension from their families [53]. Notably, the guidelines for OPS from the BC Centre of Disease Control explicitly state that youth accessing HR services and using substances is not a sufficient reason to be reported to the Ministry of Child and Family Development (the ministry responsible for child protection in BC [88]) [73]. These guidelines align with evidence-based, trauma-informed practices. However, each health authority has regional manuals that may not execute practices in the same way. An important consideration is that Vancouver Coastal Health’s OPS manual recommends that service providers contact the Ministry of Child and Family Development for youth under 16 who are accessing services within their region [89]. Ideally, access guidelines for youth should follow the provincial protocol offered by the BC Centre of Disease Control. This includes following accurate best practice guidelines, which entails no duty to report to the Ministry of Child and Family Development based on substance use or HR access alone [73].

We recommend implementing a mandate to incorporate language formulated by the BC Centre of Disease Control into the OPS guidelines of each health authority. These guidelines should adhere to trauma-informed practices and acknowledge the autonomy and capacity of the youth accessing these services.

### Provider interactions

In this review, negative interactions with healthcare providers posed a barrier to youth accessing HR services [42, 44, 45, 47, 49, 53]. Similar research shows that healthcare providers often adopt a paternalistic approach when working with YPWUD, dismissing their perspectives and discouraging further care-seeking [68, 69, 90]. In our review, youth emphasized the transformative effect of engaging with supportive healthcare professionals [42, 44, 52]. These interactions cultivated resilience and boosted the chances of their return, with these supportive providers being revered as "saints" or "angels" [42 p.5]. This facilitator highlights the need to shift organizational culture, foster positive patient-provider relationships, and increase the quality of care delivered by youth substance use providers.

We propose involving youth as valued partners in substance use care planning, conducting curriculum and patient visit reviews to eliminate harmful biases and behaviours, and centring relationship-building and trust in provider approaches.

Distrust in services, systems, and providers is often exacerbated for Indigenous YPWUD. This heightened distrust is rooted in historical and ongoing harms linked to colonization and white supremacy [83]. Our scoping review further highlights the experiences of Indigenous YPWUD and a tendency to avoid the healthcare system to prevent instances of harassment and poor treatment [44, 45, 53, 54]. An investigation found that 84% of Indigenous respondents had experienced some form of discrimination within BC’s healthcare system [91]. Indigenous patients in Vancouver are often trivialized and discharged without treatment, especially when substance use is a patient’s primary concern [83]. Embedding cultural safety and anti-racism initiatives is crucial to ensure equitable and safe care for Indigenous peoples [92, 93]. San’yas, an anti-Indigenous racism cultural safety training, was created to build capacity among healthcare workers to deliver culturally appropriate care in BC. Although initially intended as a standardized and mandatory form of awareness-raising, this program has fallen short in numerous ways. At present, it lacks Indigenous engagement in its governance and design, it is not amalgamated with health authority training programs, and is not consistently available or uniformly funded throughout the BC healthcare system [91].

In accordance with existing recommendations, we echo the need for integration, mandatory offering, and adequate funding of Indigenous-designed anti-racism education and training programs for healthcare providers across BC’s health system. To facilitate an organizational shift, actions should transcend cultural safety training. Policy and standards should prioritize cultural safety and humility expectations into the quality, accountability, and planning functions of the health system.

### Limitations

There are five limitations of this study. First, only peer-reviewed empirical journal articles using primary data were selected for this review. Although these sources can offer valuable insights, the decision to exclude grey literature and other forms of publications in our scoping review was based on resource constraints and the need for a focused and streamlined analysis. However, we recognize the merits of including grey literature sources and envision this as an opportunity for future research. Second, the exclusion of articles not written in English was implemented to ensure a consistent and comprehensive understanding of studies. This decision was made to maintain clarity and facilitate accurate interpretation of information, aligning with the language proficiency of the researchers. However, we envision this limitation to have negligible impacts on our study given the geographic focus on BC, where English is the predominant language used by researchers. Third, only five of the 13 studies sampled participants from outside Vancouver, which may have hindered our ability to capture the full spectrum of barriers and facilitators experienced by rural and remote youth. Of the studies originating in Vancouver, eight focused on street-involved youth from the At-Risk Youth Study, potentially limiting the generalizability of findings to the broader population of YPWUD. However, this cohort is well-established and provides valuable insights into the barriers faced by the broader YPWUD population. Fourth, many studies included in this review were conducted by the same groups of researchers. A lack of diverse viewpoints can limit creativity and critical analysis, potentially resulting in a less comprehensive understanding of the research topic. We recommend conducting this scoping review again when more studies have been conducted by a diverse range of authors. Fifth, our findings may not reflect the barriers and facilitators for youth located in cities with different demographics. Only a few studies focused on the intersectional identities of race, ethnicity, and gender among YPWUD, which overlook unique experiences in accessing HR services among various subgroups. It is recommended that future research include more diverse perspectives to inform more inclusive, equitable, and evidence-based approaches to substance use care.

## Conclusion

Despite the mass expansion of HR services across the province in 2016, the need for improving accessibility among youth was apparent. Various barriers hinder youth from engaging in and maintaining HR care, including challenges linked to self-stigma, navigating services, service delivery, and negative interactions with providers. Nevertheless, supportive social networks, meeting basic needs, and positive provider interactions can facilitate sustained access to HR care among youth. This review has identified and explored critical policy implications in support of YPWUD, thereby illustrating key approaches to ensuring this population receives better access to HR services and support across BC.

## Supplementary Information

Supplementary Material 1.

## Data Availability

All data analyzed during this study are listed in Table 2.

## Abbreviations

BC: British Columbia
HR: Harm reduction
OAT: Opioid agonist therapy
OPS: Overdose prevention sites
PRISMA-ScR: Preferred reporting items for systematic reviews and meta-analyses extension for scoping reviews
YPWUD: Young people who use drugs
