# Supplementary material for: A scoping review on barriers and facilitators to harm reduction care among youth in British Columbia, Canada
Source: Harm Reduct J. 2024 Oct 23;21:189. doi: 10.1186/s12954-024-01063-1 (PMC11520168; doi:10.1186/s12954-024-01063-1)
Supplement: Supplementary file 1 — Supplementary Material 1. [file 12954_2024_1063_MOESM1_ESM.docx]

**Database search strings and number of results**

**MEDLINE**

| **#** | **Search statement** | **Results** |
| --- | --- | --- |
| **1** | "youth" OR "adolescen*" OR "young adult" | 2,889,106 |
| **2** | "adolescent"[MESH] | 2,226,545 |
| **3** | ("youth" OR "adolescen*" OR "young adult") OR ("adolescent"[MESH]) | 2,889,106 |
| **4** | "substance-related disorders"[MESH] | 312,992 |
| **5** | "harm reduction"[MESH] | 4,191 |
| **6** | "harm reduction" OR "overdose prevention" OR "supervised consumption" OR "injection site" or "opioid agonist" | 29,618 |
| **7** | (("harm reduction" OR "overdose prevention" OR "supervised consumption" OR "injection site" or "opioid agonist") OR ("harm reduction"[MESH])) OR ("substance-related disorders"[MESH]) | 334,652 |
| **8** | "barrier*" OR "constraint*" OR "limitation*" OR "concern*" OR "access*" OR "experienc*" | 3,622,444 |
| **9** | "British Columbia*" OR "Vancouver" | 138,492 |
| **10** | (((("youth" OR "adolescen*" OR "young adult") OR ("adolescent"[MESH])) AND ((("harm reduction" OR "overdose prevention" OR "supervised consumption" OR "injection site" or "opioid agonist") OR ("harm reduction"[MESH])) OR ("substance-related disorders"[MESH]))) AND ("barrier*" OR "constraint*" OR "limitation*" OR "concern*" OR "access*" OR "experienc*")) AND ("British Columbia*" OR "Vancouver") | 390 |
| **11** | (((("youth" OR "adolescen*" OR "young adult") OR ("adolescent"[MESH])) AND ((("harm reduction" OR "overdose prevention" OR "supervised consumption" OR "injection site" or "opioid agonist") OR ("harm reduction"[MESH])) OR ("substance-related disorders"[MESH]))) AND ("barrier*" OR "constraint*" OR "limitation*" OR "concern*" OR "access*" OR "experienc*")) AND ("British Columbia*" OR "Vancouver") Filters: **from 2016 - 2023** | 251 |

**Scopus**

| **#** | **Search statement** | **Results** |
| --- | --- | --- |
| **1** | TITLE-ABS-KEY ( "youth" OR "adolescen*" OR "young adult" ) | 3,354,103 |
| **2** | TITLE-ABS-KEY ( "substance-related disorders" OR "harm reduction" OR "overdose prevention" OR "supervised consumption" OR "injection site" OR "opioid agonist" ) | 163,696 |
| **3** | TITLE-ABS-KEY ( "barrier*" OR "constraint*" OR "limitation*" OR "concern*" OR "access*" OR "experienc*" ) | 10,149,757 |
| **4** | TITLE-ABS-KEY ( "substance-related disorders" OR "harm reduction" OR "overdose prevention" OR "supervised consumption" OR "injection site" OR "opioid agonist" ) AND ( "barrier*" OR "constraint*" OR "limitation*" OR "concern*" OR "access*" OR "experienc*" ) | 66,272 |
| **5** | ( "British Columbia*" OR "Vancouver" ) | 628,507 |
| **6** | TITLE-ABS-KEY ( "substance-related disorders" OR "harm reduction" OR "overdose prevention" OR "supervised consumption" OR "injection site" OR "opioid agonist" ) AND ( "barrier*" OR "constraint*" OR "limitation*" OR "concern*" OR "access*" OR "experienc*" ) AND ( "British Columbia*" OR "Vancouver" ) | 3,641 |
| **7** | TITLE-ABS-KEY ( ( ( "youth" OR "adolescen*" OR "young adult" ) AND ( "substance-related disorders" OR "harm reduction" OR "overdose prevention" OR "supervised consumption" OR "injection site" OR "opioid agonist" ) ) AND ( ( "barrier*" OR "constraint*" OR "limitation*" OR "concern*" OR "access*" OR "experienc*" ) ) ) AND ( "British Columbia*" OR "Vancouver" ) | 548 |
| **8** | TITLE-ABS-KEY ( ( ( ( "youth" OR "adolescen*" OR "young adult" ) AND ( "substance-related disorders" OR "harm reduction" OR "overdose prevention" OR "supervised consumption" OR "injection site" OR "opioid agonist" ) ) AND ( ( "barrier*" OR "constraint*" OR "limitation*" OR "concern*" OR "access*" OR "experienc*" ) ) ) AND ( "British Columbia*" OR "Vancouver" ) ) AND PUBYEAR > 2015 AND PUBYEAR < 2024 | 107 |

**PsycINFO**

| **#** | **Search statement** | **Results** |
| --- | --- | --- |
| **1** | ( "youth" OR "adolescen*" OR "young adult" )OR ("adolescent"[MESH]) | 707,377 |
| **2** | ("harm reduction" OR "overdose prevention" OR "supervised consumption" OR "injection site" or "opioid agonist") OR ("harm reduction"[MESH])) OR ("substance-related disorders"[MESH]) | 10,950 |
| **3** | ("barrier*" OR "constraint*" OR "limitation*" OR "concern*" OR "access*" OR "experienc*") | 1,452,225 |
| **5** | ( "British Columbia*" OR "Vancouver" ) | 34,315 |
| **6** | ("harm reduction" OR "overdose prevention" OR "supervised consumption" OR "injection site" or "opioid agonist") OR ("harm reduction"[MESH])) OR ("substance-related disorders"[MESH]) AND ("barrier*" OR "constraint*" OR "limitation*" OR "concern*" OR "access*" OR "experienc*") | 4,563 |
| **7** | ( ( "youth" OR "adolescen*" OR "young adult" )OR ("adolescent"[MESH]) ) AND ( ("harm reduction" OR "overdose prevention" OR "supervised consumption" OR "injection site" or "opioid agonist") OR ("harm reduction"[MESH])) OR ("substance-related disorders"[MESH]) ) AND ( ("barrier*" OR "constraint*" OR "limitation*" OR "concern*" OR "access*" OR "experienc*") ) | 856 |
| **8** | ( ( "youth" OR "adolescen*" OR "young adult" )OR ("adolescent"[MESH]) ) AND ( ("harm reduction" OR "overdose prevention" OR "supervised consumption" OR "injection site" or "opioid agonist") OR ("harm reduction"[MESH])) OR ("substance-related disorders"[MESH]) ) AND ( ("barrier*" OR "constraint*" OR "limitation*" OR "concern*" OR "access*" OR "experienc*") ) AND ( ( "British Columbia*" OR "Vancouver" ) ) | 67 |
|  | ( ( "youth" OR "adolescen*" OR "young adult" )OR ("adolescent"[MESH]) ) AND ( ("harm reduction" OR "overdose prevention" OR "supervised consumption" OR "injection site" or "opioid agonist") OR ("harm reduction"[MESH])) OR ("substance-related disorders"[MESH]) ) AND ( ("barrier*" OR "constraint*" OR "limitation*" OR "concern*" OR "access*" OR "experienc*") ) AND ( ( "British Columbia*" OR "Vancouver" ) ) **Limiters** - Publication Year: 2016-2023 | 40 |
